# A Framework for Including Family Health Spillovers in Economic Evaluation

**DOI:** 10.1177/0272989X15605094

**Published:** 2016-02

**Authors:** Hareth Al-Janabi, Job van Exel, Werner Brouwer, Joanna Coast

**Affiliations:** Health Economics Unit, School of Health and Population Sciences, University of Birmingham, UK (HA, JC); Institute of Health Policy & Management, Erasmus University, Rotterdam, Netherlands (JVE, WB)

**Keywords:** economic evaluation, extra-welfarism, family, informal care, spillovers

## Abstract

Health care interventions may affect the health of patients’ family networks. It has been suggested that these “health spillovers” should be included in economic evaluation, but there is not a systematic method for doing this. In this article, we develop a framework for including health spillovers in economic evaluation. We focus on extra-welfarist economic evaluations where the objective is to maximize health benefits from a health care budget (the “health care perspective”). Our framework involves adapting the conventional cost-effectiveness decision rule to include 2 multiplier effects to internalize the spillover effects. These multiplier effects express the ratio of total health effects (for patients and their family networks) to patient health effects. One multiplier effect is specified for health benefit generated from providing a new intervention, one for health benefit displaced by funding this intervention. We show that using multiplier effects to internalize health spillovers could change the optimal funding decisions and generate additional health benefits to society.

Economic evaluation plays a growing role in the allocation of resources in the health care sector.^1^ It provides a way of maximizing a specific objective, normally health benefits, from a health care budget.^2–5^ Health maximization is achieved through evaluating health care interventions in terms of their incremental costs and incremental health benefits. Typically, health benefits are only considered in relation to patients in an economic evaluation. This neglects any wider health benefits of interventions (“health spillovers”).

Health spillovers may occur for a variety of reasons. Health care may benefit family carers, for example, by relieving them of emotionally and physically demanding caring responsibilities.^6^ Treating a patient’s health problems may also reduce family members’ anxiety^7,8^ and alleviate feelings of grief.^9^ Furthermore, patients are embedded in social networks, where individuals’ outcomes are codependent on one another.^10^ As a result, changes in care for one individual may have health-related consequences for a wider social network around the patient.

Frameworks to account for spillovers in economic evaluation have—to date—focused on the *welfare* effects of health care. Culyer’s work in the 1970s and 1980s developed the concept of the caring externality.^11–13^ In these articles, Culyer shows how public intervention in health care may internalize a caring externality and improve social welfare. Basu and Meltzer^14^ developed a family utility function to model the wider welfare effects of health care interventions. They show how the value of treatment increases when the altruism between family members is taken into account. In addition, a number of studies have empirically estimated the welfare effects on parents and partners from illness and intervention.^15–18^

Applied economic evaluation, however, frequently takes an extra-welfarist position. Here the objective is not to maximize utility but some other socially relevant objective, normally health output.^3,19^ This requires the identification, measurement, and valuation of all health effects stemming from the intervention.^3^ Authors have argued for the need to consider health of carers^4,20,21^ and other individuals in the patients’ network^8,22^ in economic evaluation. However, recent reviews have found only a handful of applied economic evaluations consider carers’ health in addition to patients’ health.^23,24^ By ignoring health effects on carers and others in the broader family network, economic evaluations may misrepresent the health benefits arising from health care interventions. However, it is presently unclear as to how such health spillovers can be included in a consistent and rigorous manner in an economic evaluation.

In this article, we develop a conceptual framework for including health spillovers in “extra-welfarist” economic evaluation, where the focus is on maximizing health benefits from a fixed health care budget. This corresponds with a commonly applied “health care perspective” for economic evaluation,^3^ requested by health technology assessment agencies in many countries (e.g., the United Kingdom, Canada, New Zealand, and Brazil^1^). Specifically, we investigate 1) how conventional practice in economic evaluation can be modified to take health spillovers into account and 2) when such spillovers would ultimately matter in terms of funding decisions and the resulting health benefits. We apply this framework to account for family health effects arising from interventions. The term *family* is used loosely to describe the close network of individuals around the patient. The focus is on health effects that arise from social and psychological mechanisms, separate from infectious disease (pathogenic) transmission. Health effects generated through reduced disease transmission would usually already be considered in an economic evaluation as they accrue to potential patients; such effects are not the focus of this study.

The article is conceptual in nature but also draws on data from a study of the long-term family impact of meningitis to illustrate key issues. The second section derives decision rules to explicitly account for health spillovers in economic evaluation. The third and fourth sections present data on health spillovers to illustrate when health spillovers matter in health care funding decisions. The fifth section discusses issues in including health spillovers in economic evaluation.

## Maximizing Health in the Presence of Spillovers: A Theoretical Approach

### Conceptual Framework

In this article, we focus on economic evaluation where the underlying objective is to maximize the stream of health benefits from a given health care budget. Economic evaluation in this form is typically conducted with the decision rule stated in expression 1. This states that the intervention is recommended on efficiency grounds if the ratio of incremental costs (Δ*c*) to incremental health benefits (Δ*h*) is less than some threshold ratio (*k*), representing the opportunity costs of diverting resources to the proposed intervention.^2^ All notation is listed in Table 1. If the left-hand side of expression 1 is less than the right-hand side, then the provision of the intervention is judged to be efficient, in the sense that it is likely to generate more health benefits than it displaces.

ΔcΔh<k.

**Table 1:** Notation for the Terms Used in the Decision Rules for Economic Evaluation

Symbol	Definition
Δ*c*	Incremental health care costs of the proposed intervention
Δ*h*	Incremental health benefits of the proposed health care intervention
Δ*h_p_*	Incremental health benefits to patients of the proposed health care intervention
Δ*h_dp_*	Incremental health displaced to patients of the proposed health care intervention
*k*	Cost-effectiveness threshold (£ per unit of displaced health benefit)
*k_p_*	Cost-effectiveness threshold (£ per unit of displaced health benefit to patients)
Δ*c_d_*	Incremental health care costs displaced by the proposed intervention
Δ*h_n_*	Incremental health benefits to patients’ network members of the proposed health care intervention
Δ*h_dn_*	Incremental health displaced to patients’ network members of the proposed health care intervention
*m*	Multiplier effect on the stream of patient health benefits
*m_i_*	Multiplier effect on the stream of patient health benefits generated by intervention
*m_d_*	Multiplier effect on the stream of patient health benefits displaced by intervention

As highlighted earlier, it is common practice that only health benefits to *patients* are measured and valued in applied economic evaluation. We therefore suggest that the way in which the decision rule is operationalized in practice is represented in expression 2. Here incremental health benefits and the threshold ratio have a subscript *p* because we assume that both are estimated in terms of patient health generated and patient health forgone. We assume the opportunity cost of diverting resources is measured in terms of patient health forgone, as this is the assumption underlying the most comprehensive recent work to estimate a cost-effectiveness threshold.^25^

ΔcΔhp<kp.

As argued earlier, health spillovers are likely to be present in the health system. In order words, health care interventions may result in health consequences to patients’ family networks in addition to the patients themselves. This means that in reality incremental health benefits (Δ*h*) are the sum of incremental health benefits to patients (Δ*h_p_*) and patients’ family networks (Δ*h_n_*). At this stage, we make no assumption about the scale (or even direction) of such health spillovers, only that they may be nonzero.

### Generating and Displacing Spillover Effects

Within a system with a fixed budget, funding a new health care intervention implies displacing health sector activity elsewhere. Just as it is possible that a new intervention could generate health spillovers, so it follows that displacing activity elsewhere in the health system may also displace health spillovers. For example, withdrawing a beneficial intervention may result in additional anxiety to patients’ family members.

To take account of health spillovers in a consistent manner, both health spillovers generated and those displaced need to be considered. This means both sides of expression 2 need to be modified. On the left-hand side, this can be done by explicitly accounting for the health spillovers generated by the intervention in the denominator (i.e., representing Δ*h* as (Δ*h_p_ +* Δ*h_n_*)). On the right-hand side, *k* represents the ratio of displaced costs (Δ*c_d_*) and displaced benefits (Δ*h_d_*), and so the right-hand side can be amended by expanding the denominator to include displaced health spillovers (see expression 3).

Δc(Δhp+Δhn)<(Δcd)(Δhdp+Δhdn).

Since *k_p_* = Δ*c_d_ /*Δ*h_dp_, k_p_* can be substituted back into expression 3 to express the decision rule in terms of the information conventionally used in an economic evaluation and the additional information needed to represent the presence of health spillovers (see expression 4).

Δc(Δhp+Δhn)<kp*hdp(Δhdp+Δhdn).

### Specifying Multipliers

The decision rule in expression 4 implies that health spillovers generated (Δ*h_n_*) and displaced (Δ*h_dn_*) by each decision would have to be directly measured. In practice, measurement of all health spillovers generated and displaced may not be practical, if only because it often remains implicit what interventions get displaced. In such cases, health spillovers might still be accounted for in the decision rule by specifying a “multiplier” (*m*) to represent the ratio of incremental total health benefits (comprising health benefits to patients and their family networks) to incremental patient health benefits from the intervention. This multiplier requires only the ratio of spillover benefits to patient benefit to be known (rather than the absolute magnitude of spillover benefits). This multiplier can be specified for health benefits generated by a new intervention (*m_i_*) and those displaced (*m_d_*) (see expressions 5 and 6).

mi=(Δhp+Δhn)Δhp.

md=(Δhdp+Δhdn)Δhdp.

Rearranging expression 4 and substituting the 2 multipliers generates expression 7. This specifies a decision rule for incorporating health spillovers in a way that clearly relates to the conventional decision rule (expression 2).

$$\left(\frac{\Delta c}{\Delta h_{p}}\right)*\left(\frac{m_{d}}{m_{i}}\right)<k_{p}.$$

This means that a decision rule for economic evaluation that incorporates health spillovers can be expressed in terms of the conventional information (incremental costs, incremental patient health benefits, and a threshold ratio based on patient health displaced) and 2 multiplier effects. One multiplier effect refers to health benefits generated by the health care intervention (*m_i_*) and the other to health benefits displaced by the health care intervention (*m_d_*).

### Implications for Maximizing Health Output in the Presence of Spillovers

**Figure 1:**
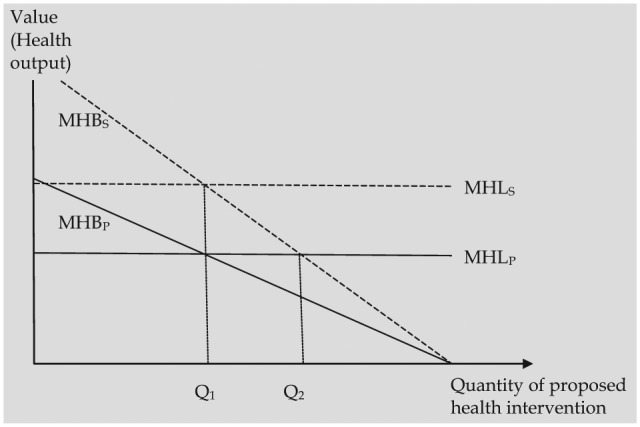
Health benefits to society and health benefits to patients are maximized at the same point (Q_1_) when spillovers are constant across a new intervention and displaced health care. MHB_P_, marginal health benefits to patients; MHB_S_, marginal health benefits to society; MHL_P_, marginal health losses to patients; MHL_S_, marginal health losses to society.

In this section, we graphically illustrate the effect of different-sized spillovers on resource allocation decisions. We consider a national decision to fund a new health care intervention. In this situation, the benefits of the intervention, in terms of patient health, can be represented graphically by the marginal health benefits (MHB_P_) schedule in Figure 1. As with a conventional demand curve for a normal good, this schedule declines with increasing quantity to reflect the fact that some patients benefit more from the intervention than others.

The opportunity cost of funding the intervention, in terms of foregone patient health benefits elsewhere, is depicted by the marginal health losses (MHL_P_) schedule. This is constant on the assumption that the opportunity cost per unit does not vary with each unit of the intervention (i.e., the intervention cost is small compared with the health care budget as a whole). Under these conditions, using information on patient health benefits to maximize health benefits results in funding the intervention up to Q_1_. Here the marginal benefits (in terms of health generated) and costs (in terms of health displaced) are equal.

#### Constant spillovers

The presence of health spillovers means that the marginal health benefits generated by the intervention to individuals in society as a whole (MHB_S_) exceed the marginal health benefits to patients. (In this example, we have assumed that health spillovers are proportional to patient health effects [within a given intervention]. Other formulations of health spillovers are possible, but these do not materially alter the conclusions of the analysis in this section.)

Turning to the MHB_S_ schedule, we can see this intersects the MHL_p_ schedule at Q_2_. This appears to suggest that health benefits (which include patient health benefits and health spillovers) would be maximized at a somewhat higher level of funding (Q_2_). This point equates marginal health benefits to society with marginal health displaced to patients (MHL_p_). However, this fails to take into account the opportunity costs, in terms of displaced health spillovers. If the displaced health spillovers are of the same proportion (relative to patient health) as those generated by the new intervention, this results in an upward shift in the MHL_P_ schedule to MHL_S_ (representing the marginal health losses to society from displaced health care spending). The MHB_s_ and MHL_s_ schedules intersect at Q_1_ and the population health maximization equilibrium is reestablished at quantity Q_1._ In this example, incorporating additional information on health spillovers in decision making results in the same equilibrium as when the focus is only on patient health benefit. In other words, even if health spillovers are present, if they are of a fixed proportion relative to patient health benefits across the new and displaced interventions, then decision making based on *patient* health benefits is sufficient to maximize net health benefits to the population. This finding is also implied by expression 7 (see “Specifying Multipliers”), which indicates that when the ratio of health multipliers is 1, the conventional decision rule will result in health-maximizing decisions.

#### Relatively large and small spillovers

**Figure 2:**
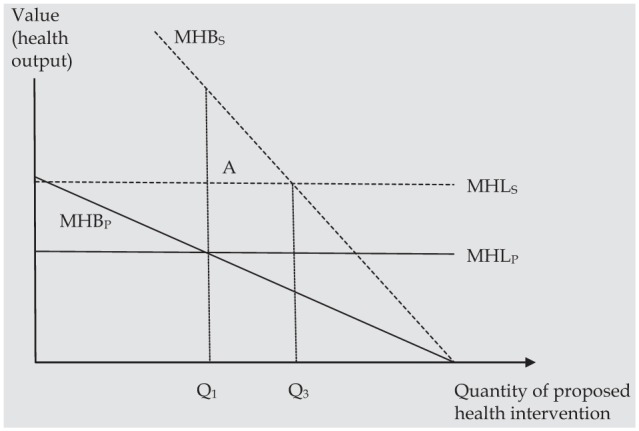
When spillovers on a new intervention are “large,” health benefits to society are maximized at a higher quantity (Q_3_) than when patient health alone is considered (Q_1_). Triangle A represents the additional health benefit to society from increasing the quantity of the intervention from Q_1_ to Q_3_.

What if health spillovers are not constant across new and displaced health care interventions? In Figure 2, health spillovers from the proposed intervention are large relative to those displaced by the intervention. The optimal position for generating health output would be to increase the funding of the intervention up to Q_3_ where MHB_S_ and MHL_S_ are equal. This may in practice represent widening access to an intervention, beyond the patient group that derives the most benefit. Expanding funding to Q_3_ would result in additional health benefits to society, represented by triangle A, relative to decisions based on patient health benefits alone.

**Figure 3:**
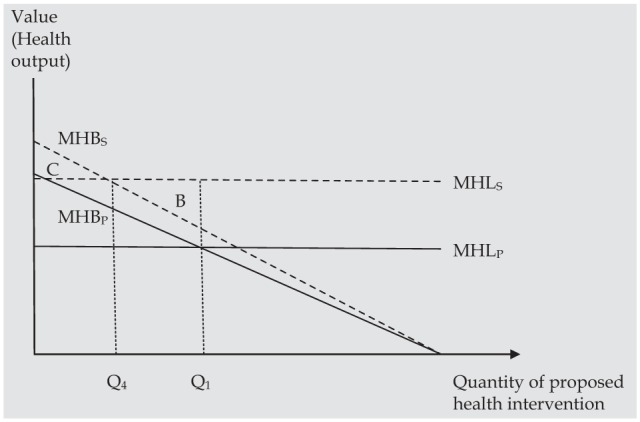
When spillovers on a new intervention are “small,” health benefits to society are maximized at a lower quantity (Q_4_) than when patient health alone is considered (Q_1_). Triangle B represents the health benefit to society from reducing the quantity of the intervention from Q_1_ to Q_4_. Triangle C represents the net health benefit to society generated at Q_4_.

In Figure 3, health spillovers from the proposed intervention are small relative to those displaced by the intervention (albeit still positive). Taking into account the marginal health benefit to society (MHB_S_) and the marginal health losses to society (MHL_S_) implies a lower level of funding to maximize health output (Q_4_). In this case, a decision to scale back the provision of the health care intervention from Q_1_ to Q_4_ would result in additional health benefits to the population equal to triangle B. This example demonstrates a somewhat counterintuitive finding that it could be optimal to scale back, or withdraw, an intervention even if, in some cases, the intervention generates positive health spillovers. In fact, if triangle B is larger than the triangle bounded by the vertical axis and the MHB_S_ and MHL_S_ schedules (triangle C), it implies that net health benefits would be higher if the proposed intervention was not funded at all relative to being funded all the way up to Q_1_. This is because the value of the health spillovers that are generated is more than offset by the value of health spillovers that are displaced. Taken together, Figure 2 and Figure 3 illustrate the implication of expression 7, that if health spillovers (and hence multipliers) are not constant across the health system, then explicitly incorporating health spillovers in the decision-making process is necessary to maximize health benefits.

## Are Health Spillovers Likely to Vary Across Intervention Contexts?

As documented in “Maximizing Health in the Presence of Spillovers,” health spillovers matter for economic evaluation when they vary across health care interventions. In this section, we illustrate the potential for variation in spillovers by using data from study of family impact of meningitis. Meningitis is an acute illness, but it can result in a wide variety of disabling after-effects (conditions) after the initial recovery. These conditions include behavioral problems, learning difficulties, hearing and sight loss, seizures, and amputations. These conditions provide some insight into the spillover effects on family members’ health status and therefore whether the potential spillover benefits of treatment and prevention may vary across conditions.

This section draws on data gathered from an earlier study to examine whether meningitis affected family members’ lives.^26^ The earlier study indicated that conditions associated with meningitis were, in general, associated with reduced health status for both survivors (henceforth “patients”) and their family (henceforth “carers”). In the present investigation, we analyzed the association between the presence of individual conditions and 1) the health status of patients and 2) the health status of carers. We then analyzed whether the pattern of associations differed between patients and carers. This suggests whether multipliers are likely to be constant across treatments for the different disabling conditions arising from meningitis.

To determine associations between individuals’ health status and the conditions associated with meningitis, we ran 2 simple ordinary least squares (OLS) regression models: one for patients and one for their carers. For both groups, health status was measured using the EQ-5D-5L^27^ and scored using interim value sets for the United Kingdom.^28^ In the regression models, we controlled for individuals’ age, sex, and the time elapsed since the initial infection, as these factors may confound the relationship between after-effects and health status. The results of the regression models are presented in the online appendix.

**Figure 4:**
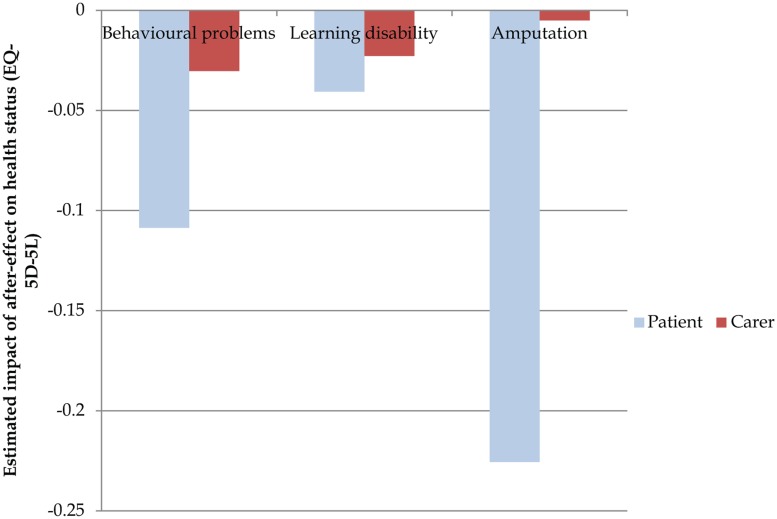
Mean implied health losses (on EQ-5D-5L scale) from after-effects of meningitis incurred by patients and their carers.

In Figure 4, we have drawn on the regression results to highlight the marginal impact of three common conditions on the current health status of patients and their carers. The figure suggests that conditions affect differentially the health status of patients and their carers. For patients, amputations have the biggest mean impact (–0.23), followed by behavioral problems (–0.11) and then mild/moderate learning disability (–0.04). However, for carers, behavioral problems have a bigger negative impact (–0.03) than amputations (–0.01).

Table 2 shows the mean annual health benefit (in terms of EQ-5D-5L) for patients and carers that would, in theory, result from treating or preventing each condition. The implied multiplier effects of treatment are listed next in the table. These are calculated based on the formula listed in expression 4 and are intended to be illustrative only. We have calculated 2 possible multiplier effects representing scenarios where health spillovers extend to 1 or 2 carers per patient. We have assumed additivity in the multiplier effect as the multiplier represents the ratio of total health effects (calculated by adding effects on patients and family members) to health effects on patients. The findings in Table 2 show that the multipliers differ across the 3 conditions. The multiplier on treating behavioral problems is relatively large, and the multiplier on treating amputations is relatively small. Furthermore, the multipliers diverge from one another as more family members (carers) are assumed to be affected by the health spillovers. In reality, health spillovers will be likely to tail off with increased social distance from the patient,^26^ limiting this effect somewhat.

**Table 2:** Health Losses from Selected After-Effects of Meningitis and the Implied Health Multiplier Effects from Intervening to Treat/Prevent the After-Effects

Patient After-Effect	Mean Impact of After-Effect (on EQ-5D-5L)		Multiplier (*m_i_*)	
	On Patient Health Status	On Carer Health Status	One Network Member Affected	Two Network Members Affected
Behavioral problems	−0.109	−0.030	1.28	1.56
Mild or moderate learning disability	−0.041	−0.023	1.56	2.12
Amputation(s)	−0.226	−0.005	1.02	1.04

## Internalizing Health Spillovers

### Using Family Health Spillovers to Inform Resource Allocation: An Example

In this section, we extend the analysis to illustrate the potential impact of health spillover information on funding decisions and health benefits. We analyze decisions relating to the provision of a set of 6 hypothetical interventions to treat the 3 conditions (behavioral problems, learning difficulties, and amputations) highlighted in the previous section. For each condition, we specify a relatively more cost-effective intervention (A) and a relatively less cost-effective intervention (B). We assume that in reality, each health care intervention generates health benefits for patients and 2 carers, although the scenarios differ in terms of whether the decision maker is aware of this spillover information.

The 6 interventions are packages of care costing £2 million each. The patient health benefits specified are hypothetical but deliberately selected to generate cost-effectiveness ratios that are reasonably near to a threshold value. We assume that the opportunity cost to patient health of providing these £2 million packages of care is 100 units of health benefits (corresponding with a threshold of £20,000 per unit of health). We also assume an opportunity costs to carers’ health of 16 units of health for every £2 million of spending. This spillover of 0.16 is illustrative but is based on figures showing the potential health spillover of conditions in other contexts.^8,29^

To examine the impact of explicitly incorporating health spillover information in the decision-making process, we consider 3 decision-making scenarios. In the first scenario, funding decisions are based on maximizing net health benefits to patients only. In the second scenario, decisions are based on maximizing health benefits to patients and a single carer. In the third scenario, decisions are based on maximizing health benefits to patients and 2 carers. Information on the degree of health spillover is taken from the previous section.

### Decision Making with Different Information on Health Spillovers

In scenario 1, we assume that to free up £2 million to fund an intervention, we displace 100 units of health elsewhere in the health care sector. The optimal response to maximize net health benefits in scenario 1 is therefore to fund any of the £2 million intervention packages that generate more than 100 units of health benefit. Applying this decision rule in scenario 1 results in both interventions A and B for amputations and intervention A for behavior being funded (Table 3).

**Table 3:** Perceived Health Benefits and Funding Decisions under Different Information Scenarios

	Scenario 1^a^		Scenario 2^b^		Scenario 3^c^	
	Treatment A	Treatment B	Treatment A	Treatment B	Treatment A	Treatment B
Behavioral problems	**120**	80	**154**	102	**187**	125
Mild/moderate learning disability	80	70	**125**	109	**170**	**148**
Amputation(s)	**120**	**110**	**122**	112	125	114

Note: Interventions that would be recommended for funding on the basis of maximizing (perceived) health benefits are in bold.

a. Decisions are based on maximizing health benefits to patients only (i.e., adopt treatments generating >100 units of health benefit).

b. Decisions are based on maximizing health benefits to patients and to a single carer (i.e., adopt treatments generating >116 units of health benefit).

c. Decisions are based on maximizing health benefits to patients and 2 carers (i.e., adopt treatments generating >132 units of health benefit).

In scenario 2, the decision maker incorporates information on health spillovers extending to a single carer for each patient. In terms of the proposed interventions, the multipliers (considering only a single carer) listed in Table 2 have therefore been applied to the patient health benefits in scenario 1. To reflect the displaced health spillovers to a single carer, we have used a displacement multiplier of 1.16 (see “Using Family Health Spillovers to Inform Resource Allocation: An Example”). The new decision rule is therefore to adopt interventions that generate more than 116 units of health benefit. Under the new information, the pattern of funding changes so that intervention A for learning disability should now be funded, and intervention B for amputations should no longer be funded to maximize health benefits.

In scenario 3, we assume that the decision maker has full information of the health benefits generated by the intervention (i.e., to the patient and both carers). This can be demonstrated by revising the multipliers applied to health benefits generated and displaced by funding decisions. To reflect the displaced health spillovers to 2 carers, the multiplier on displaced health benefits is now assumed to be 1.32. The new decision rule is therefore to adopt interventions that generate more than 132 units of health benefit. In scenario 3, we see that using all information about the health spillovers leads to a further intervention being funded (intervention B for learning disability) and another intervention that is no longer funded (intervention A for amputations).

This is a stylized example, but it does illustrate 3 important points. First, that in the presence of spillovers that vary across conditions, decisions based on patient health benefits can be far from optimal. In this example, decisions based on patient health benefits alone secured less than one-third (30/109) of the potential net health benefits that could be secured from optimal decision making. (Net health benefits using patient benefit alone = (187 – 132) + (125 – 132) + (114 – 132) = 30. Net health benefits using patient and spillover information = (187 – 132) + (170 – 132) + (148 – 132) = 109.) Second, it is important to consider health spillovers displaced by funding decisions as well as those generated. Failing to reduce the cost-effectiveness threshold in scenario 3 (i.e., assuming *m_d_* is equal to 1) would have resulted in all interventions being adopted (losing an additional 32 units of health relative to the optimal position). Third, considering only a single carer when health spillovers in reality extend further (as in scenario 2) may only capture part of the extra stream of health benefits possible from using spillover information.

## Discussion

This article outlines a technique to incorporate health spillover effects on family members in economic evaluation. We suggest health spillovers could be incorporated through the estimation of 2 multiplier effects: one relating to health benefits generated and one relating to health benefits displaced by a new intervention. This represents a relatively simple modification of the existing decision rule for maximizing health output. Furthermore, by introducing a multiplier for health displaced, as well as health generated, this prevents bias toward adopting new interventions at the cost of existing interventions in health technology appraisal.

Health spillovers become more relevant for economic evaluation when the 2 multipliers diverge from one another. This may happen when a new intervention is associated with particularly large health spillovers; for example, a new health care intervention may alleviate substantial strain on patients’ carers and family members. However, it is equally important to consider contexts where a new intervention might generate negligible or even negative health spillover impacts. In these cases, inference based on health benefits to patients will result in *overprovision* of the intervention. Negative health spillovers may occur when health care interventions result in harmful spillovers in the wider population, as, for example, in the case of antibiotic resistance.^30,31^ They may also occur when interventions shift the burden of care onto families, potentially to the benefit of patients but to the detriment of family members’ health. There is also some evidence of positive spillovers of caring.^32,33^ Presumably, such spillovers would be lost if the intervention prevented the illness and hence caring responsibilities. However, in general, evidence suggests that illness results in negative health spillovers on family networks.^6,34,35^

The multiplier effect will also be influenced by the size of the network affected by the health care intervention. Large multipliers may be present when there is a wide group of people surrounding each individual patient, even if the health effects on individuals are relatively small and these individuals are not providing informal care.

Our framework differs in several respects from a previous theoretical approach to incorporating family spillovers developed by Basu and Meltzer.^14^ In our approach, we assume that a societal decision maker, such as a government or agency, makes health care funding decisions to maximize health benefits to the population they serve. In contrast, Basu and Meltzer take as their starting point an individual who makes decisions about purchasing medical care based on maximizing his or her own utility. Thus, the frameworks differ in both the decision maker and the decision maker’s objective. Furthermore, Basu and Meltzer explicitly model the probabilities of individuals surviving and being married because the decision maker is an individual as opposed to an organization. Finally, in contrast to some empirical studies,^15,35^ we estimate spillovers using a health status measure scored using social values, rather than estimating spillovers using directly elicited utilities. This offers consistency with the way in which health effects are typically valued for patients^36^ and avoids conflating health and nonhealth arguments in the maximand.

Our assertion that health spillovers and multiplier effects are likely to vary by context is consistent with the broader literature. Evidence indicates that some health conditions have a disproportionately large impact on the family.^35^ For example, mental health conditions are often associated with adverse outcomes for family members,^34,37–39^ childhood health problems may place particular emotional strain on parents,^29,34,40^ and end-of-life care can have important ramifications for the health of family members.^9^ Furthermore, spillover effects are not necessarily only a function of the illness. Contexts involving substantial informal care may result in greater multiplier effects because of the dual effects of physically demanding care and emotional worry for family members.^7,8^

In this study, we focused on economic evaluation where the objective is maximizing health output from scarce health care resources. Other maximization objectives for economic evaluation are possible too,^19^ including maximizing subjective well-being,^41^ maximizing capability gains,^42^ and, more conventionally in mainstream economics, maximizing the consumption value of health care.^2^ In all these cases, the explicit inclusion of spillovers effects, whether health, well-being, or welfare, is relevant to properly maximizing the objective function, provided one takes account of spillovers in the opportunity costs too.^43^ Indeed, where the objective is the maximization of the consumption value of health care, a number of authors have already highlighted the importance of considering spillovers (or “caring externalities”) in determining the economically optimal level of health care provision in society.^11,14,17,30^ Within the realm of health maximization, health spillovers might be conceived as “health externalities” as they represent socially relevant, third-party impacts of resource allocation decisions that are not internalized in the decision-making process.

This article focuses on the inclusion of “outcome” spillovers in economic evaluation, but a couple of points are worth considering in relation to “cost” spillovers. First, there is some evidence to suggest that illness may affect the health care use of the patient’s family.^44^ This suggests that treating or preventing illness may result in cost savings in treating patients’ family networks. These spillovers ought to be included, in theory, where a health care perspective is taken. Second, under a societal perspective, additional cost spillovers are important to consider. These comprise the productivity (and time) losses associated with the illness for patients and their family members, out-of-pocket costs, and any costs or savings to other public and private agencies.^3^

In certain contexts, there may be ethical concerns in accounting for health spillovers in resource allocation decisions. For example, the inclusion of health spillovers may imply greater funding for services for people with dependents at the expense of those without.^14^ However, it is also important to note that the *exclusion* of health spillovers implies these health benefits have zero social value: a position that has not been subjected to normative scrutiny. Going forward, it may be helpful to distinguish between the analysis inherent in maximizing an objective function (whether health or otherwise) and the political process surrounding health care funding decisions. In the former, it is important to explicitly account for health spillovers to identify which interventions *do* in fact maximize health output. To inform the political process of making a health care funding decision, such spillovers might be presented alongside patient health benefits as well as in aggregated form to enable decision makers with different normative stances to weight them differently.^20,21^

In practice, including health spillover information routinely in economic evaluations is likely to be challenging. Determining multiplier effects in different contexts is likely to require a combination of measurement and modeling work. In the short-term, data will be needed on the impact of health care interventions on the health of patients’ family members. In the longer term, it might be quite acceptable to model multipliers in certain decision contexts without the need to resort to primary data collection. However, this requires more information about the size of affected networks, how health spillovers vary across disease contexts, and whether they persist over time. Health technology assessment bodies could potentially play their part by ensuring that, where relevant, health spillovers are not seen as an “optional extra” but an integral part of understanding the impact of funding decisions on population health. In some contexts, this is starting to be done; for example, in their 2013 guidance, the National Institute for Health and Care Excellence (in England and Wales) states that economic evaluations should consider “all direct health effects, whether for patients or, when relevant, carers.”^45^ However, it is important to bear in mind that health spillovers can extend to the wider family network beyond those who self-identify as “carers.”

In conclusion, to pursue a goal of health maximization, economic evaluations need to incorporate health spillovers in addition to patient health benefits. We have proposed a framework for doing this through the inclusion of multipliers applied to health benefit gained and displaced by a new intervention. This will add some extra complexity to economic evaluation. However, inclusion of health spillovers has the potential to not just improve the conduct of economic evaluation but also to include the family perspective in decision making and generate real health benefits to the population.

## Supplementary Material

Supplementary material
