# Supplementary material for: A Framework for Including Family Health Spillovers in Economic Evaluation
Source: Med Decis Making. 2016 Feb;36(2):176–86. doi: 10.1177/0272989X15605094 (PMC4708618; doi:10.1177/0272989X15605094)
Supplement: Supplementary material [file DS_10.11770272989X15605094.pdf]

DOI: 10.1177/0272989X15605094

Appendix: Regression models of current health status of patients (model 1) and carers (model 2) in the context of meningitis after after-effects

Variables	MODEL 1<br>Patient health status<br>regression coefficients (95% CI)	MODEL 2<br>Carer health status<br>regression coefficients (95% CI)
**After effects**		
Behavioural problems	-0.11 (-0.14 to -0.08)***	-0.03 (-0.05 to -0.01)**
Learning disability (mild)	-0.04 (-0.07 to -0.01)**	-0.02 (-0.05 to 0.00)
Learning disability (severe)	-0.22 (-0.34 to -0.09)***	-0.08 (-0.16 to 0.01)
Speech/language	-0.05 (-0.09 to 0.00)*	-0.02 (-0.05 to 0.02)
Hearing loss (one ear)	-0.02 (-0.05 to 0.02)	0.01 (-0.02 to 0.04)
Hearing loss (both ears)	-0.06 (-0.10 to -0.02)**	-0.01 (-0.04 to 0.03)
Sight loss	-0.02 (-0.08 to 0.05)	-0.03 (-0.09 to 0.03)
Other visual impairment	-0.03 (-0.09 to 0.02)	-0.02 (-0.06 to 0.02)
Seizures	-0.06 (-0.14 to 0.01)	-0.03 (-0.09 to 0.03)
Hydrocephalus	-0.08 (-0.19 to 0.03)	0.05 (-0.02 to 0.12)
Hypotonia	-0.13 (-0.20 to -0.05)**	-0.04 (-0.09 to 0.01)
Motor limitations	-0.19 (-0.28 to -0.09)***	0.01 (-0.06 to 0.08)
Incontinence	-0.18 (-0.28 to -0.08)***	0.02 (-0.02 to 0.06)
Balance problems	-0.05 (-0.09 to -0.01)*	-0.02 (-0.06 to 0.01)
Pain (after medication)	-0.12 (-0.18 to -0.06)***	-0.05 (-0.09 to -0.01)*
Amputation(s)	-0.23 (-0.30 to -0.15)***	-0.01 (-0.05 to 0.04)
Scarring or tissue damage	-0.02 (-0.05 to 0.01)	-0.02 (-0.05 to 0.01)
Abnormal bone growth	-0.08 (-0.14 to -0.01)*	0.01 (-0.03 to 0.05)
Arthritis/severe joint pain	-0.08 (-0.12 to -0.03)**	-0.04 (-0.07 to 0.00)
Kidney damage	-0.01 (-0.08 to 0.05)	-0.01 (-0.07 to 0.05)
Other	-0.06 (-0.09 to -0.03)***	-0.02 (-0.05 to 0.00)
**Control variables**		
Time since infection (yrs)	0.002 (0.000 to 0.004)*	-0.001 (-0.002 to 0.001)
Survivor age (yrs)	-0.003 (-0.004 to -0.002)***	Not included
Network member age (yrs)	Not included	-0.003 (-0.004 to -0.002)***
Survivor sex (male)	0.031 (0.007 to 0.055)*	Not included
Network member sex (male)	Not included	0.027 (0.004 to 0.051)*
	R^2 = 0.646	R^2 = 0.141
	n=936	n=936

* p<0.05, **p<0.01, ***p<0.001
